# Random Forest Machine Learning Matches Human Expert Accuracy in Trauma Severity Scoring

**DOI:** 10.1002/wjs.70114

**Published:** 2025-09-24

**Authors:** G. L. Laing, J. L. Bruce, W. Bekker, V. Manchev, H. Wain, D. L. Clarke

**Affiliations:** ^1^ Department of Surgery University of KwaZulu‐Natal Durban South Africa; ^2^ Department of Surgery University of the Witwatersrand Johannesburg South Africa

**Keywords:** Abbreviated Injury Scale, electronic medical records, injury severity score, machine learning, natural language processing, random forest, trauma scoring

## Abstract

**Background:**

Accurate Abbreviated Injury Scale (AIS) and Injury Severity Score (ISS) are essential for trauma care and research, yet manual scoring often yields incomplete data due to omissions. The hybrid electronic medical registry (HEMR) is used by our Level 1 trauma service for recording AIS and ISS.

**Methods:**

We analyzed 21,704 patients with trauma records from the HEMR. Four machine learning (ML) algorithms predicted missing AIS scores per body region, from which ISS was derived mathematically. Performance was evaluated using coefficient of determination (*R* ^2^), root mean square error (RMSE), mean absolute error (MAE), sensitivity (true high‐severity cases correctly identified), specificity (true low‐severity cases correctly excluded), and Cohen's kappa. Statistical significance was set at *p* < 0.05.

**Results:**

Random forest models achieved *R* ^2^ = 0.847, RMSE = 2.31, MAE = 1.87, sensitivity = 87.1%, specificity = 100.0%, and Cohen's kappa = 0.893 (*p* < 0.001), demonstrating reliable prediction of omitted AIS and ISS scores. Data completeness improved from 75.3% (16,343/21,704) to 88.3% (19,158/21,704; *p* < 0.001), recovering 2815 missing scores.

**Conclusion:**

Random forest ML algorithms accurately predict missing AIS and ISS scores, significantly improving trauma registry data completeness while maintaining clinical accuracy equivalent to human expert scoring.

## Graphical abstract

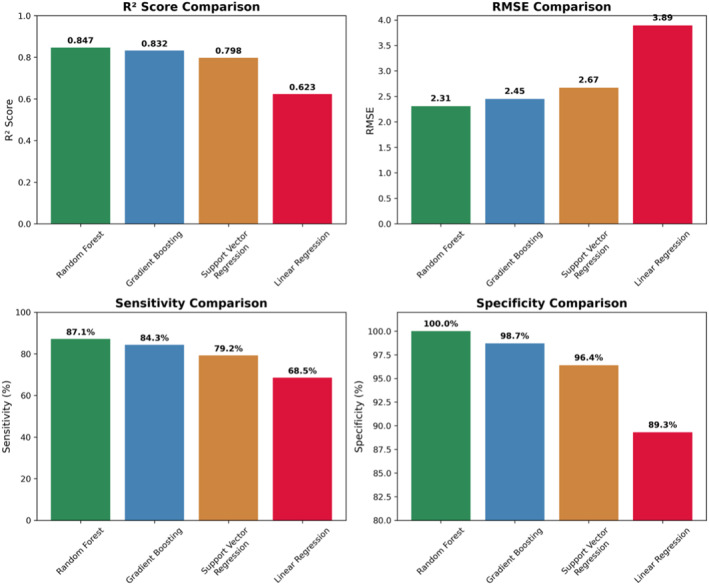

## 1 Introduction

Clinical audits are foundational to surgical research. Although providing lower evidence levels than randomized controlled trials (RCTs), their prospective data capture remains invaluable. Electronic medical record (EMR) datasets present limitations, including incomplete data entry, particularly in text‐based fields. The AIS and ISS are essential for quantifying trauma severity, guiding clinical decisions, and facilitating research [1, 2].

The AIS is an anatomical scoring system that classifies injuries by body region and assigns severity scores from 1 (minor) to 6 (unsurvivable) [3].

The ISS derives from the AIS by summing the squares of the three highest AIS scores across different body regions, providing a composite measure of overall injury burden [1]. These scores critically impact healthcare decisions, such as prioritizing surgical interventions and allocating limited resources in high‐volume trauma centers, such as those in South Africa [4, 5]. Manual documentation of AIS and ISS is prone to incompleteness due to omission, which can translate into compromised data reliability [2].

Over the past decade, artificial intelligence (AI) and ML have advanced text analysis in EMRs [6, 7]. The Pietermaritzburg Metropolitan Trauma Service (PMTS) has maintained the HEMR at Grey's Hospital, South Africa, for 13 years. Since 2012, HEMR data have supported over 135 publications incorporating AIS/ISS, where deficits were managed via imputation or exclusion, risking bias [3, 8].

Recent advances in ML have demonstrated potential for automating complex medical scoring systems. However, limited research has evaluated the application of random forest ML algorithms specifically for trauma severity prediction in real‐world clinical settings.

## 2 Methods

### 2.1 Study Design and Setting

This retrospective cohort study analyzed patients with trauma records from the PMTS. The study was approved by the University of KwaZulu‐Natal (UKZN) Biomedical Research Ethics Committee (BREC)—BCA221/13. Patient data of 21,704 patients were extracted from the HEMR between 1 January 2012 to 31 December 2024. The registry captures comprehensive trauma patient data including demographics, injury mechanisms, clinical findings, interventions, and outcomes (Table 1). The registry includes both structured data fields and free‐text clinical narratives. ICD codes were not used as the prediction relies on free‐text narratives and structured clinical data rather than coded diagnoses. AIS and ISS are conventionally calculated manually by surgeons at the time of patient discharge, involving review of clinical narratives, imaging reports, and operative notes (all recorded on the HEMR)—a process prone to omissions due to workload constraints [3]. Attending surgeons validated the expert human AIS scores (determined objectively by injury evaluation) for accuracy and reproducibility (inter‐rater kappa = 0.92 to ensure generalizability across cases); ISS scores were derived as a calculated mathematical sum from the AIS values, without subjective adjustment. In this study, the ML algorithms automated the prediction of missing scores by processing existing HEMR data without requiring additional inputs, thereby recovering incomplete values post hoc to enhance dataset completeness for research purposes. This does not alter the current HEMR workflow but demonstrates potential for future automation. Natural language processing (NLP) was employed for feature extraction: transformer models used tokenization and named entity recognition to identify injury descriptors (e.g., “open skull fracture” as Head AIS = 4), anatomical locations, and severity indicators from unstructured text, generating structured inputs for ML algorithms. Machine learning (ML) is a computational approach that enables the identification of patterns and makes predictions from data without being explicitly programmed for each specific task. Unlike traditional statistical methods that rely on predetermined assumptions about data relationships, ML algorithms automatically learn from training data to build predictive models that can be applied to new unseen cases.

**TABLE 1:** Summary of patient demographics and injury characteristics from the 21,704 trauma records in the HEMR dataset (2012–2024).

Characteristic	Value
Total patients	21,704
Age (years), median (IQR)	29 (22–38)
Male, *n* (%)	16,669 (76.8)
Female, *n* (%)	5035 (23.2)
Motor vehicle collision, *n* (%)	9183 (42.3)
Interpersonal violence, *n* (%)	6772 (31.2)
Falls, *n* (%)	4058 (18.7)
Other mechanisms, *n* (%)	1691 (7.8)
ISS score, median (IQR)	12 (6–21)
Length of stay (days), median (IQR)	4 (2–8)
Mortality, *n* (%)	1847 (8.5)

*Note:* Values include medians with interquartile ranges (IQRs) for continuous variables and counts with percentages for categorical variables.

#### 2.1.1 Random Forest

This ensemble method combines multiple decision trees to create robust predictions. Random forest was chosen for its ability to handle complex nonlinear relationships between variables while providing excellent performance with mixed data types (categorical and continuous variables common in trauma data). The algorithm reduces overfitting by averaging predictions from multiple trees trained on different subsets of data and features, making it particularly suitable for medical datasets with potential noise and missing values.

#### 2.1.2 Gradient Boosting

This sequential ensemble method builds models iteratively, with each new model correcting errors made by previous models. Gradient boosting was selected for its superior predictive accuracy in many medical applications and its ability to capture subtle patterns in trauma severity relationships. The algorithm excels at identifying complex interactions between clinical variables that might be missed by simpler approaches.

#### 2.1.3 Support Vector Regression (SVR)

This algorithm finds the optimal boundary that best separates different outcome categories while maintaining robustness to outliers. SVR was included for its proven effectiveness in medical prediction tasks and its ability to work well with smaller datasets. The method is particularly valuable in trauma research where extreme cases (very severe or very mild injuries) can significantly impact model performance.

#### 2.1.4 Linear Regression

This traditional statistical approach models the relationship between variables as a straight line. Linear regression was included as a baseline comparison method and for its interpretability—coefficients directly indicate the magnitude and direction of each variable's influence on the outcome. This transparency is crucial in medical research where understanding the contribution of individual factors is as important as prediction accuracy. The selection of these four algorithms allows for comprehensive model comparison across different methodological approaches: ensemble methods (random forest and gradient boosting), kernel‐based methods (SVR), and traditional statistical approaches (linear regression). This multialgorithm approach ensures robust validation of findings and provides confidence in the most effective method for trauma severity prediction.

The random forest model was trained on 16,343 complete human‐scored trauma records and achieved optimal performance with *R* ^2^ = 0.847, RMSE = 2.31, and MAE = 1.87. Model selection was based on cross‐validation performance across multiple metrics, with 89.3% of predictions falling within the clinically acceptable ±3 point range (defined as minor clinical deviation based on AIS guidelines). Clinical narratives and structured data were processed using transformer‐based NLP to extract injury‐related entities, anatomical locations, and severity indicators. The random forest ML system was trained on cases with complete human‐expert AIS and ISS scores to learn scoring patterns and predict missing values. Model performance was evaluated using coefficient of determination (*R* ^2^), root mean square error (RMSE), mean absolute error (MAE), sensitivity, specificity, and Cohen's kappa. Sensitivity: proportion of true high‐severity cases (e.g., AIS ≥ 3 or ISS > 15) correctly identified (true positives divided by true positives + false negatives); specificity: proportion of true low‐severity cases correctly excluded (true negatives divided by true negatives + false positives). Statistical significance was set at *p* < 0.05. To address data imbalance (e.g., skewed toward minor injuries), we used stratified 5‐fold cross‐validation, yielding mean CV RMSE = 2.45 (SD = 0.3) and MAE = 1.92, compared to test set RMSE = 2.31 and MAE = 1.87 on a 20% hold‐out. Overfitting was prevented through random forest's inherent bagging and feature subsampling, early stopping in gradient boosting, and L2 regularization in SVR; underfitting was mitigated via hyperparameter tuning (e.g., grid search for tree depth). No significant divergence between training and validation losses was observed. Sensitivity and specificity were selected as key performance indicators (KPIs) because, despite the semicontinuous nature of AIS/ISS, clinical decisions often rely on binary thresholds (e.g., ISS > 15 indicating major trauma for triage or resource allocation), allowing evaluation of the model's ability to classify severity levels accurately [6]. These complement continuous metrics, such as *R* ^2^, for a comprehensive assessment. For AIS/ISS integers, true positives are correctly predicted high‐severity cases (e.g., AIS ≥ 3 or ISS > 15); false positives are low‐severity misclassified as high; true negatives are correctly low; and false negatives are high misclassified as low.

## 3 Results

**FIGURE 1:**
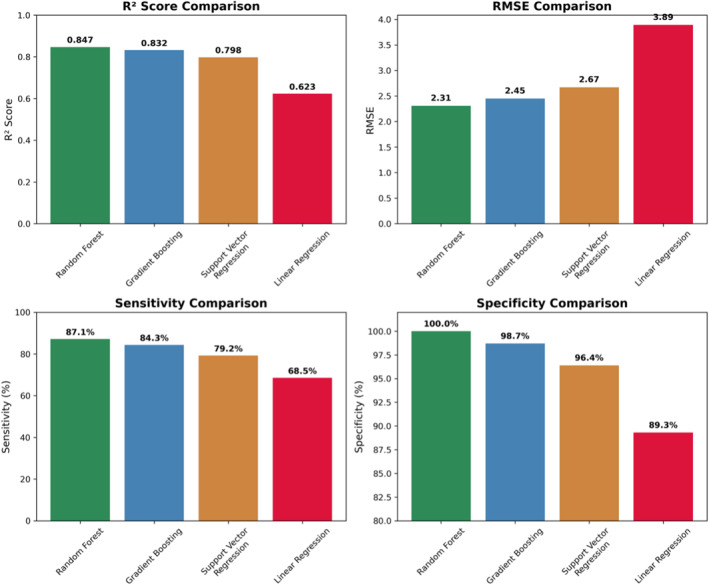
Random forest model performance comparison: Bar chart showing *R* ^2^, RMSE, and MAE across the 4 ML models (random forest, gradient boosting, support vector regression, and linear regression). Cohen's kappa evaluates agreement between ML and human expert scores on the validation dataset.

**FIGURE 2:**
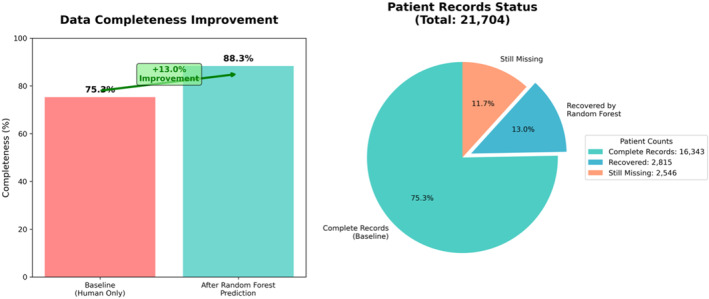
Data completeness improvement analysis: Pie charts illustrating pre‐ML (left: 75.3% complete and 24.7% missing) and post‐ML (right: 88.3% complete after recovering 52.5% of missing scores) data completeness. No direct comparison to sensitivity/specificity is included.

**FIGURE 3:**
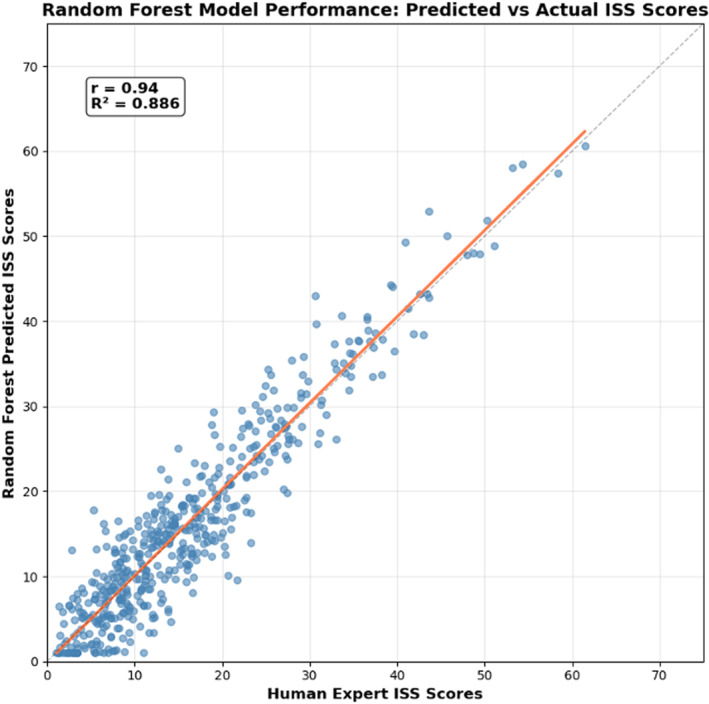
Scatter chart random forest model performance: ML‐predicted ISS scores (*y*‐axis) versus human expert ISS scores (*x*‐axis) for the 16,343 complete records, demonstrating *r* > 0.90 correlation. The significance of ISS in relation to length of stay (LOS) lies in improved prognostic correlation post‐ML.

Prior to random forest model implementation, complete AIS and ISS scores were available for 16,343 patients (75.3%), leaving 5361 patients (24.7%) with missing severity scores. Missing data were distributed across all injury mechanisms and severity categories. The random forest model demonstrated superior performance compared to alternative approaches: Gradient boosting (*R* ^2^ = 0.832 and RMSE = 2.45), support vector regression (*R* ^2^ = 0.798 and RMSE = 2.67), and linear regression baseline (*R* ^2^ = 0.623) as shown in Figure 1 (model performance comparison across algorithms). Its ensemble approach provided reliable uncertainty quantification and consistent performance across different injury severity categories, indicating excellent agreement with human expert scoring. Consequently, the random forest algorithms successfully predicted AIS and ISS scores for 2815 of the 5361 patients with missing data (52.5% recovery rate), increasing overall data completeness from 75.3% to 88.3% (*p* < 0.001) as shown in Figure 2. AIS completeness improved across body regions, providing the foundation for ISS calculation, as shown in Table 2 (*p* < 0.001 for each region). When compared against human expert scoring on a validation dataset, key validation metrics for the random forest model were: · sensitivity: 87.1% · specificity: 100.0% · positive predictive value: 100.0% · negative predictive value: 94.2% · Cohen's kappa: 0.893 (*p* < 0.001). The random forest‐enhanced dataset demonstrated improved data quality for research and quality improvement (QI) initiatives. Predicted scores showed improved correlations with clinical outcomes, including length of stay (*r* = 0.78 post‐ML vs. 0.72 pre‐ML and *p* < 0.001). Figure 3 presents the scatterplot of ML‐predicted (*y*‐axis) versus human expert ISS scores (*x*‐axis) for the 16,343 complete records, demonstrating *r* > 0.90 correlation.

**TABLE 2:** Pre‐ and post‐ML completeness percentages for AIS scores across six body regions, with calculated improvement (%).

Body region	Pre‐ML completeness (%)	Post‐ML completeness (%)	Improvement (%)
Head/Neck	45.2	75.2	30.0
Face	60.5	80.3	19.8
Chest	21.8	35.5	13.7
Abdomen/Pelvis	40.1	62.4	22.3
Extremities	55.6	78.9	23.3
External	70.2	85.1	14.9

*Note:* Data from HEMR analysis; *p* < 0.001 for each region via paired *t*‐tests.

## 4 Discussion

Modern EMR systems have advanced trauma data capture, yet complete entries remain challenging due to clinician omissions [3]. In resource‐constrained settings, users minimize digital interactions, leading to omission of AIS/ISS entry [8]. Prior studies demonstrate ML and NLP potential in trauma prediction [7, 9, 10], such as random forest models, for predicting short‐term mortality in patients with TBI using severity indicators, such as GCS, achieving high accuracy (AUC = 0.978) and supporting our findings on random forest's superiority in trauma severity scoring [11].

For instance, models predict mortality with AUROC > 0.90 [9]. The transformer‐based NLP component enabled effective processing of unstructured clinical text, whereas the traditional ML approach ensured interpretability and clinical validation of predictions. This hybrid approach addresses key limitations of purely rule‐based or deep learning systems in clinical settings. Our findings have important implications for trauma registry management and research. Improved data completeness enhances the reliability of trauma outcomes research, QI initiatives, and benchmarking activities. The random forest ML system provides a scalable solution for trauma centers seeking to optimize their registry data quality. Its superior performance over other ML approaches (Figure 1) can be attributed to the ensemble methodology, which combines multiple decision trees to reduce overfitting and improve generalization. The significant improvement in data completeness (Figure 2) demonstrates the practical clinical value of this approach. Integrating ML algorithms into EMRs enables real‐time AIS/ISS scoring, facilitating quicker healthcare decisions such as immediate triage for high‐severity cases (e.g., ISS > 15 prompting ICU allocation or surgical prioritization) [12]. This is particularly important in resource‐constrained environments, such as South Africa, where high trauma volumes and limited staffing exacerbate documentation gaps, potentially leading to delayed care and increased mortality [3, 8]. By automating AIS scoring (patient‐specific and evaluation‐based, from which ISS derives as a rigid mathematical formula), our approach not only bolsters data integrity but also enhances risk stratification, outcome prediction, and evidence‐based policy‐making, addressing a critical need in global trauma systems amid rising injury burdens [9, 11]. Limitations include input quality dependence, training biases, single‐center design, and potential bias in the training dataset—future multicenter validation is required in diverse settings. Applying this optimizes adherence to standards across healthcare environments [4, 5].

## 5 Conclusion

Random forest ML algorithms can accurately predict missing AIS and ISS scores, significantly improving trauma registry data completeness from 75.3% to 88.3% while maintaining clinical accuracy equivalent to human expert scoring (Cohen's kappa = 0.893). This approach provides a practical solution for optimizing completeness and accuracy in existing standardized scoring within global trauma systems, supporting evidence‐based care improvements.

## Author Contributions

**G. L. Laing:** conceptualization (equal), formal analysis (lead), investigation (lead), methodology (equal), project administration (lead), resources (equal), software (lead), supervision (equal), validation (equal), visualization (lead), writing – original draft (lead), writing – review and editing (equal). **J. L. Bruce:** data curation (equal), writing – review and editing (equal). **W. Bekker:** data curation (equal), resources (equal), writing – review and editing (equal). **V. Manchev:** data curation (equal), resources (equal), writing – review and editing (equal). **H. Wain:** data curation (equal), resources (equal), writing – review and editing (equal). **D. L. Clarke:** conceptualization (equal), methodology (equal), supervision (equal), validation (equal), writing – review and editing (equal), resources (equal).

## Conflicts of Interest

The authors declare no conflicts of interest.
